# Predictive models of digestible and metabolizable energy of wheat in growing pigs

**DOI:** 10.3389/fnut.2025.1662909

**Published:** 2025-10-14

**Authors:** Chuanxin Shi, Qingyan Zhu, Zhuangzhuang Lu, Yongqi Yang, Yanan Ding, Junmin Li, Chunmei Zhang, Li Xi, Xiaofang He, Zhiqiang Li, Jincheng Han, Guangli Yang, Yan Guo, Bingbing Ma

**Affiliations:** ^1^Department of Animal Science, College of Smart Animal Husbandry, College of Biology and Food, Shangqiu Normal University, Shangqiu, China; ^2^Henan University-Enterprise Research and Development Center for Biological Feed, Shangqiu, China; ^3^College of Animal Science and Food Engineering, Jinling Institute of Science and Technology, Nanjing, China

**Keywords:** digestible energy, metabolizable energy, pig, prediction equation, wheat

## Abstract

**Background:**

Wheat is progressively utilized in animal husbandry. However, the chemical properties of wheat are influenced by breed and climate, consequently impacting its economic value and application in feed formulation. This study was conducted to evaluate the digestible energy (DE) and metabolizable energy (ME), as well as apparent total tract digestibility (ATTD) of nutrients in 17 samples of wheat for growing pigs.

**Methods:**

Fifty-one growing barrows were randomly allotted to 17 experimental diets (3 pigs in each diet). The experiment included two successive periods, and per period lasted for 12 days, including 7 days for diet adaptation, 5 days for urine and feces collection.

**Results:**

There was significant (*p* < 0.01) difference in DE content of 17 wheat cultivars. The DE and ME contents ranged from 16.06 to 16.88 MJ/kg DM and 15.65 to 16.36 MJ/kg DM, respectively. The coefficients variation (CV) of the chemical compositions were bulk density (3.14%), crude protein (CP) (9.00%), neutral detergent fiber (NDF) (34.60%), acid detergent fiber (ADF) (9.65%) and ether extract (EE) (10.16%) in the 17 wheat samples. Except for the ATTD of phosphorus (P), there were significant differences in the ATTD of gross energy (GE) (*p* = 0.01), organic matter (OM) (*p* = 0.02), CP (*p* < 0.01), NDF (*p* < 0.01), ADF (*p* < 0.01), EE (*p* = 0.04) and calcium (Ca) (*p* = 0.03) in 17 wheat cultivars.

**Conclusion:**

DE was significantly positively correlated with ME and CP content, GE was significantly negatively correlated with starch, and CP content was positively correlated with GE, DE and ME contents. Based on statistical analysis of chemical components of wheat samples, the GE, CP, starch, and bulk density are key variables for predicting effective energy, and the most suitable regression equations of DE and ME in different wheat cultivars were DE = 26.6394 − 0.6783 GE (MJ/kg) + 0.1618 CP (%) and ME = −0.3869 + 0.7788 DE (MJ/kg) + 0.0336 starch (%) + 0.0020 bulk density (g/L).

## 1 Introduction

Energy feed is a standard raw material in pig complete formula feed, accounting for no less than 60% of complete formula feed composition. Corn, as the energy source of pig complete formula feed, is the primary representative raw material of energy feed. However, China’s annual wheat imports have exceeded 20 million metric tons over the past five years. This growing dependency on imported corn underscores the urgency for identifying alternative energy sources to enhance feed security and reduce production costs. Wheat is one of the three major cereals, second only to corn and rice. With extensive cultivation area and high yield, wheat wholly or partially replaces corn as an energy feed for pigs, which can reduce production costs and alleviate pressure on feed resource shortages. Based on current market conditions, the price of wheat is typically RMB 50–100 per metric ton lower than that of corn. Furthermore, the higher protein content of wheat (wheat: 14–15%; corn: 8–9%) allows for a reduction of about 1–2% in soybean meal usage, leading to an additional cost saving of RMB 30–40 per metric ton (1). The application of wheat could reduce approximately 55% of the corn and 7% of the soybean meal in corn-soybean diet of pig (2). However, the chemical properties of wheat are influenced by breed and climate, different varieties and areas caused differences of chemical properties of wheat and affected utilization of nutrients by pigs (3, 4). Therefore, it is pivotal to evaluate the accurate energy values of different wheat cultivars.

Although wheat can replace corn economically, the variability in wheat nutritional components poses the main obstacle to reasonable utilization. The chemical composition of wheat is influenced by genetic diversity, environmental conditions, and processing technics. These variations significantly impact the energy value and nutrient availability of wheat for pigs (5). Specifically, factors such as cultivar type, growing season, soil quality, and climatic conditions can lead to substantial variability in key nutritional components including crude protein (CP), starch, neutral detergent fiber (NDF), acid detergent fiber (ADF), and gross energy (GE). Therefore, it is essential for facilitating the extensive utilization of wheat in the pig diet to accurately evaluate available energy content. Precision optimization of feed formulations is carried out according to the digestive and metabolic utilization rates of feed components.

Determination of the effective energy of feed ingredients is time-consuming and labor-intensive based on animal digestion or metabolism trials, and unable to effectively avoid errors caused by factors, such as different varieties, batches, and cultivation regions (6). Moreover, traditional methods fail to account for the dynamic changes in nutrient composition caused by genetic improvements and evolving agronomic techniques. Therefore, establishing prediction equations of effective energy serves as a practical approach to address these challenges according to determination of the chemical components and variations in feed ingredients of different sources. The prediction equations is always utilized to evaluate the digestible energy (DE) and metabolizable energy (ME) values of feed ingredients on account of its advantages of saving cost and improving detection efficiency based on its chemical and physical properties (7). Such predictive equations would enable rapid, economic, and accurate energy assessment, facilitating optimal dietary formulation and utilization of wheat in pig production systems.

We hypothesized that different wheat cultivars exhibited significant variation in energy values and nutrient digestibility. Therefore, the object of the experiment was to evaluate the DE, ME and the apparent total tract digestibility (ATTD) of nutrients in 17 wheat cultivars, and build the equations of DE and ME according to their chemical constituents and physical characteristics.

## 2 Materials and methods

### 2.1 Wheat samples information

The yield of wheat in Henan Province is the highest in the world. Henan province (China) is located at 30 to 36 degree of north latitude, which belongs to warm temperate transition continental monsoon climate and is suitable for wheat growth. A total of 17 wheat samples were collected from 16 cities in Henan Province during mid-to-late May at the fully ripe stage (Table 1). The chemical constituents of 17 wheat cultivar samples are analyzed and presented in Table 2.

**Table 1:** Information of 17 wheat samples.

Items	Wheat cultivars	Collection site (Henan province)	Items	Wheat cultivars	Collection site (Henan province)
1	Zhongyu 12	Nanyang City	10	Aibai 987	Xiangcheng City
2	Huapei 1	Shangqiu City	11	Bainong 207	Yongcheng City
3	Pingan 8	Kaifeng City	12	Xinong 585	Luoyang City
4	Hefeng 3	Zhumadian City	13	Bainong 418	Jiaozuo City
5	Yangmai 15	Xinyang City	14	Zhengmai 369	Xinxiang City
6	Pingmai 02–16	Zhumadian City	15	Aifeng 68	Hebi City
7	Zhengmai 3596	Luohe City	16	Aikang 58	Anyang City
8	Luomai 26	Zhoukou City	17	Jinlun 988	Puyang City
9	Xinmai 26	Changge City			

**Table 2:** Chemical composition of 17 wheat samples (%, DM basis).

Samples^1^	Items^2^										
	Bulk density, g/L	GE, MJ/kg	DM %	CP %	Starch %	NDF %	ADF %	Ash %	EE %	Ca %	P %
1	684.00	18.62	87.81	16.82	64.62	20.99	2.81	1.93	1.62	0.10	0.47
2	740.80	18.62	88.28	15.74	64.27	24.37	2.54	1.87	1.66	0.07	0.38
3	715.90	18.58	88.14	15.73	65.10	24.20	2.78	1.95	1.51	0.07	0.50
4	711.40	18.69	88.03	15.70	63.35	27.38	2.80	1.99	1.46	0.08	0.50
5	666.70	18.31	88.15	11.26	67.54	16.72	3.00	1.94	1.93	0.10	0.47
6	713.70	18.52	88.40	13.88	65.10	29.20	2.94	1.99	1.69	0.10	0.46
7	734.20	18.69	88.11	16.09	64.08	19.32	2.58	1.87	1.39	0.08	0.36
8	750.70	18.57	88.59	15.26	62.63	23.31	3.33	2.11	1.54	0.11	0.47
9	722.40	18.80	89.08	16.85	59.49	12.57	2.61	1.77	1.87	0.06	0.35
10	735.30	18.58	89.02	16.46	63.96	10.82	2.41	1.96	1.75	0.10	0.48
11	722.90	18.69	88.87	17.63	64.21	11.65	2.44	1.91	1.71	0.10	0.41
12	723.20	18.53	88.41	15.34	64.59	12.50	2.36	1.94	1.93	0.09	0.42
13	737.80	18.42	88.72	16.18	64.22	13.74	2.76	1.90	1.53	0.08	0.44
14	754.00	18.57	88.42	16.01	60.42	12.95	2.43	1.96	1.62	0.09	0.46
15	749.80	18.47	88.56	15.38	63.64	14.43	2.57	1.89	1.47	0.09	0.39
16	733.90	18.65	88.87	15.49	63.19	13.78	2.73	1.85	1.52	0.08	0.35
17	684.00	18.60	90.16	15.36	63.43	11.05	2.33	1.91	1.75	0.10	0.41
Mean	724.80	18.58	88.57	15.60	63.76	17.59	2.67	1.93	1.64	0.09	0.43
CV	3.14	0.94	0.62	9.00	2.81	34.60	9.65	3.72	10.16	16.91	11.56

1 The information about sources of wheat is described in Table 1.

2 GE, gross energy; DM, dry matter; CP, crude protein; NDF, neutral detergent fiber; ADF, acid detergent fiber; EE, ether extract; Ca, calcium; P, phosphorus.

### 2.2 Animal and experimental design

The procedures of this experiment were verified and approved by the Institutional Animal Care and Use Committee of Shangqiu Normal University (2023-1109). This experiment was executed in the animal experimental base of Shangqiu Normal University (Shangqiu, China).

Fifty-one Duroc × (Landrace × Yorkshire) growing barrows (body weight of 30.1 ± 1.8 kg) were cultivated in temperature-controlled room, and environment temperature was ranged from 20 to 25 °C. Each pig was cultivated in metabolism crates (1.4 × 0.7 × 0.6 m). Barrows were randomly allotted to an 17 × 2 incomplete Latin Square design with 17 diets and two consecutive 12-d periods. Each diet included 3 barrows in each period with 6 replications per diet. Dietary composition was shown in Table 3. The chemical constituents of diets were measured and showed in Table 4. Pigs received an equal amount of feed equivalent to 4% of body weight, and feed and water were available ad libitum. The diet ingredient were ground by a 2.5-mm screen (hammer mill). The feed was divided into two equal portions and administered at 8:00 h and 16:00 h daily. The experiment was lasted for 12 d (7 d for adaptive phase and 5 d for samples collection). The experimental samples and protocol were based on previous studies (8). From 8 d to 12 d, feces were gathered and stored at −20 °C to suppress degradation of the samples caused by microorganism. At last, the collected samples for feces were weighed, dissolved, and then homogenized thoroughly. Feces sample of 300 g was dried at 65 °C for 72 h. The 50 mL of 6 N HCl was added into buckets to gather urine sample. We measured the volume of collected urine daily, and 20% of urine was stored at −20 °C.

**Table 3:** Ingredient composition of the experimental diets (%, as-fed basis).

Ingredient	Wheat diet
Wheat	96.50
Limestone	0.70
Dicalcium phosphate	1.90
Sodium chloride	0.30
Choline chloride	0.10
Premix^1^	0.50
Total	100.00

1 Premix provided the following per kg of complete diet: vitamin A as retinyl acetate, 5,512 IU; vitamin D_3_ as cholecalciferol, 2,200 IU; vitamin E as DL-alpha-tocopheryl acetate, 30 IU; vitamin K_3_ as menadione nicotinamide bisulfite, 2.2 mg; vitamin B_12_, 27.6 μg; riboflavin, 4 mg; pantothenic acid as DL-calcium pantothenate, 14 mg; niacin, 30 mg; choline chloride, 400 mg; folacin, 0.7 mg; thiamin as thiamine mononitrate, 1.5 mg; pyridoxine as pyridoxine hydrochloride, 3 mg; biotin, 44 μg; Mn as MnO, 40 mg; Fe as FeSO_4_·H_2_O, 75 mg; Zn as ZnO, 75 mg; Cu as CuSO_4_·5H_2_O, 100 mg; I as KI, 0.3 mg; Se as Na_2_SeO_3_, 0.3 mg.

**Table 4:** The chemical composition of experimental diets (%, as-fed basis).

Diets^1^	Items^2^									
	GE, MJ/kg	DM %	CP %	Starch %	NDF %	ADF %	Ash %	EE %	Ca %	P %
1	15.89	88.46	14.13	54.42	10.13	2.29	4.04	1.51	0.76	0.96
2	16.02	90.90	13.00	54.51	10.92	2.43	3.68	1.52	0.79	0.93
3	15.93	88.66	12.60	55.08	10.43	2.26	3.91	1.29	0.81	0.96
4	15.97	88.55	14.89	53.50	10.53	2.23	3.86	1.19	0.72	0.98
5	15.71	88.28	9.85	57.16	10.61	2.57	3.97	1.46	0.76	0.95
6	15.89	88.42	12.17	55.24	13.89	5.08	3.87	1.62	0.72	0.95
7	15.89	88.78	12.53	54.21	9.61	2.32	3.91	1.40	0.79	0.93
8	16.08	88.77	13.91	53.25	14.34	5.53	4.03	1.24	0.77	0.96
9	16.15	89.17	14.61	50.88	11.71	2.35	3.57	1.55	0.78	0.92
10	16.16	88.93	11.75	54.66	10.00	2.29	3.98	1.37	0.76	0.94
11	16.14	88.85	14.28	54.78	10.60	2.25	3.74	1.32	0.76	0.95
12	15.87	88.46	13.38	54.81	11.63	2.24	3.82	1.29	0.80	0.94
13	15.96	88.57	12.37	54.71	10.37	2.26	3.91	1.16	0.79	0.96
14	15.96	88.52	13.84	51.28	10.97	2.41	3.84	1.50	0.71	0.96
15	15.91	88.72	11.12	54.11	11.72	2.34	3.95	0.91	0.78	0.93
16	15.93	88.37	12.59	53.92	12.02	2.67	3.75	1.04	0.79	0.92
17	15.98	88.88	12.51	54.90	9.72	2.18	3.85	0.91	0.74	0.96

1 The information about sources of wheat is described in Table 1.

2 GE, gross energy; DM, dry matter; CP, crude protein; NDF, neutral detergent fiber; ADF, acid detergent fiber; EE, ether extract; Ca, calcium; P, phosphorus.

### 2.3 Chemical analysis

The detection of dry matter (DM), starch, CP, ash, calcium (Ca), and phosphorus (P) is carried out according to a previous report (9). The ether extract (EE) of samples was determined as previously described (10). The NDF and ADF were analyzed according to McGhee and Stein (11). The GE of all samples were analyzed by an Automatic Isoperibol Oxygen Bomb Calorimeter (Parr 1281 Calorimeter, Moline, IL).

### 2.4 Calculations

The ATTD of nutrients, DE, and ME of wheat samples were calculated according to the equation described by previous research (12):


$${\mathrm{DE}}_{d}=({\mathrm{GE}}_{i}-{\mathrm{GE}}_{f})/F_{i}$$

$${\mathrm{DE}}_{w}={\mathrm{DE}}_{d}/0.965$$

$${\mathrm{ME}}_{d}=({\mathrm{GE}}_{i}-{\mathrm{GE}}_{f}-{\mathrm{GE}}_{u})/F_{i}$$

$${\mathrm{ME}}_{w}={\mathrm{ME}}_{d}/0.965$$

$$\text{ATTD}=(\mathrm{DN}\times F_{i}-\mathrm{FN}\times F_{o})/(\mathrm{DN}\times F_{i})$$

In which DE_d_ and ME_d_ are the DE and ME in wheat diets (MJ/kg of DM); GE_i_ is the amount of GE ingested for each pig (MJ of DM); F_i_ represented the actual amount of feed intake; the amount of GE in the feces and urine of each pig (MJ of DM) are represented by GE_f_ and GE_u_; DE_w_ and ME_w_ are the content of DE and ME in each wheat sample (MJ/kg of DM); DN and FN are the concentrations of nutrients in diets and feces; F_o_ is the output of feces.

### 2.5 Statistical analysis

Data analysis was executed by SAS 9.2. A pig was an experimental unit. The diet was regarded as the fixed effect, and the period and animal were the random effects. The correlation and stepwise regression analysis between chemical compositions and DE, ME were analyzed by the CORR and REG procedures (13). The LSMEANS procedure was executed to calculate the mean values. Tukey’s multiple range test was used to separate statistical differences. The significance level was set at *p* < 0.05. The equations were considered as the best-fit prediction equations of DE and ME when the *R*^2^ is greatest and the residual standard deviation (RSD) is smallest (14).

## 3 Results and discussion

### 3.1 The chemical composition and bulk density of 17 different samples of wheat

In this study, the chemical composition and bulk density were presented in Table 2 on a DM basis. CP, starch, and crude fat are essential sources of nitrogen and energy to promote pig growth. The previous study indicated that the contents of CP, starch, and EE were ranged from 12.40 to 17.40%, 54.10 to 58.40%, 1.40 to 1.80% in 12 samples of wheat from Canada Prairie and Western (15). Zhao et al. (14) found that the contents of CP, starch, and EE were ranged from 14.25 to 16.14%, 53.43 to 57.52%, 1.30 to 1.34% in 2 samples of wheat. The results of the CP and EE contents mentioned above were consistent with our data. We found that the contents of CP and EE were ranged from 11.26 to 17.63%, 1.39 to 1.93% in 17 samples of wheat. Moreover, there was a considerable variation in the CP content within different cultivars of wheat (11.26 to 17.63%; CV = 9.00%). This indicated that although the varieties and origins were different, the variability in the energy value of wheat and its primary determinants were similar. Zhang et al. (4) reported that GE, CP, EE, NDF, and ADF is 17.24 MJ/kg, 14.07, 1.44, 15.21, 2.45% in extruded wheat, respectively. Apart from the inherent varietal differences, subsequent processing technologies (such as extrusion and pelleting) also influence the ultimate nutritional value of wheat.

In our study, higher starch content was observed compared with those of the previous reports (14, 15). The variation of wheat starch and CP contents were caused by different varieties, growing season and regions (16). Many previous studies reported that a considerable variation of CP was caused by different climatic conditions rather than soil quality or genetic factors in wheat sample (17, 18). The abundant soil moisture and low environment temperature during the ripening of the wheat results in lower content of protein, whereas the CP content of wheat was higher in the warm weather conditions (15). Starch constitutes the principal component of wheat and serves as the substrate for sugar provision during fermentation. Given the growing demand for wheat as a renewable resource, research aimed at developing wheat varieties with elevated starch content may attract keen interest (19). The content of wheat carbohydrates could provide energy and determine the pattern and efficiency of energy release (20). In addition, protein-riched wheat was preferentially utilized for breadmaking and the requirement of society (21, 22). Whereas wheat, as a feedstuff and renewable resource for biofuel production, eventually contains large amounts of starch (23).

The level of wheat fiber components was affected by the cultivars and growing environment. Many previous studies reported that NDF and ADF fractions were ranged from 16.34 to 25.10% and 2.70 to 5.10% in different cultivars and harvest times of wheat (4, 16, 24, 25). Other researches demonstrated that NDF content and ADF content were from 10.99–15.83%, 2.04–4.70%, which were consistent with our study (12, 14, 26). We found that there were large variations in the NDF concentration (10.82 to 29.20%; CV = 34.60%), and the ADF content (2.33 to 3.33%) was lower compared with the reports mentioned above. Climatic temperature has a great influence on the content of NDF and ADF of wheat. The level of fiber component in wheat in winter was lower than in summer (12). The nutrient absorption capacity of wheat root was decreased in low-temperature, moreover, part of the nutrients in wheat are used to resist cold stress (27). The above findings demonstrated that the interplay between genetic attributes and environmental factors determine nutritional composition of wheat, and ultimately results in a predictable and comparable range of available energy content for swine (see Table 5).

**Table 5:** The DE and ME of experimental diets.

Diets^1^	Items^2^					
	GE intake, MJ	FE output, MJ	UE output, MJ	DE in diet, MJ/kg	ME in diets, MJ/kg	ME/DE
1	125.36	13.08^ab^	4.15	14.23^abc^	13.70	96.26
2	131.79	14.16^ab^	3.45	14.25^abc^	13.85	97.16
3	132.97	15.40^a^	4.33	14.07^bc^	13.56	96.43
4	134.30	14.27^ab^	4.80	14.26^abc^	13.71	96.09
5	109.92	13.74^ab^	2.47	13.73^d^	13.38	97.44
6	129.66	14.94^ab^	3.00	14.05^c^	13.70	97.51
7	115.99	13.25^ab^	3.15	14.07^bc^	13.65	96.98
8	111.76	13.09^ab^	3.35	14.20^abc^	13.66	96.22
9	133.95	14.78^ab^	5.25	14.36^abc^	13.74	95.67
10	110.21	10.72^b^	3.74	14.58^a^	14.04	96.35
11	136.51	13.56^ab^	3.61	14.53^a^	14.10	97.01
12	131.75	13.46^ab^	5.45	14.24^abc^	13.61	95.57
13	122.63	11.23^ab^	3.28	14.48^ab^	14.07	97.09
14	132.07	15.34^a^	4.40	14.10^abc^	13.60	96.43
15	115.89	12.51^ab^	3.23	14.19^abc^	13.75	96.83
16	104.99	11.76^ab^	1.92	14.14^abc^	13.85	97.96
17	113.62	11.93^ab^	3.29	14.29^abc^	13.85	96.89
SEM	6.76	0.84	1.13	0.09	0.14	0.96
*p*-value	0.18	<0.01	0.81	<0.01	0.06	0.96

^a,b,c,d^Different letters in the same indicator represent significant differences, *n* = 6.

1 The information about sources of wheat is described in Table 1.

2 GE, gross energy; FE, fecal energy; UE, urine energy; DE, digestible energy; ME, metabolizable energy.

### 3.2 Differences in the energy contents and nutrients digestibility of diets

The ATTD of GE, OM, CP, NDF, ADF, EE and Ca showed significant differences (*p* < 0.05) (Table 6). And the ATTD of GE, DM, OM, CP, NDF, ADF, and EE ranged from 87.30 to 90.77%, 87.76 to 90.37%, 88.63 to 91.06%, 81.73 to 90.44%, 49.04 to 66.14%, 18.19 to 46.31%, 27.07 to 57.13% among 17 wheat cultivars, respectively. The ATTD of GE in our result was similar to previous studies, which reported that the ATTD of GE in the diets ranged from 86.58 to 90.79% in different wheat cultivars (4, 12, 15). An earlier research reported a lower ATTD of GE and DM (ranged from 76.2 to 87.0% and 79.1 to 89.4%) in 20 different wheat grains in growing pigs (8). The different ATTD of GE and nutrients were associated with various chemical composition of wheat grains grown in various areas and climates, for example, the ATTD of GE in winter wheat is higher than that of the spring (12, 28). Previous research demonstrated that the ATTD of DM, NDF, ADF, and CP ranged from 87.47–88.54%, 56.78–64.21%, 25.80–40.74%, and 83.63–87.58% (14), which were consistent with our study.

**Table 6:** The apparent total tract digestibility (ATTD) of nutrients in diets, %.

Diets^1^	Items^2^									
	GE, MJ/kg	DM %	OM %	CP %	NDF %	ADF %	Ash %	EE %	Ca %	P %
1	89.54^ab^	89.32^ab^	89.85^ab^	90.39^a^	54.84^abc^	25.52^b^	58.86^a^	51.05^ab^	53.07^ab^	68.38
2	88.98^ab^	89.22^ab^	89.88^ab^	87.12^a^	55.34^abc^	25.79^b^	50.45^abc^	45.56^ab^	54.19^ab^	68.51
3	88.30^ab^	88.06^ab^	88.78^ab^	87.88^a^	49.04^c^	18.19^b^	39.39^bcd^	43.04^ab^	53.53^ab^	67.76
4	89.33^ab^	89.15^ab^	89.77^ab^	89.60^a^	54.98^abc^	18.42^b^	43.41^abcd^	48.44^ab^	46.88^b^	66.59
5	87.43^b^	87.76^b^	88.63^b^	81.73^b^	57.01^abc^	27.85^b^	36.48^cd^	27.07^b^	49.94^ab^	66.74
6	88.45^ab^	88.50^ab^	89.13^ab^	85.95^a^	66.14^a^	46.31^a^	54.76^ab^	45.18^ab^	43.04^b^	65.47
7	88.58^ab^	88.79^ab^	89.56^ab^	85.87^a^	53.59^bc^	24.93^b^	40.32^bcd^	50.34^ab^	54.84^ab^	72.63
8	88.32^ab^	88.21^ab^	89.01^ab^	87.91^a^	65.51^a^	46.14^a^	32.10^d^	50.67^ab^	53.11^ab^	69.84
9	88.93^ab^	88.98^ab^	89.66^a^	89.93^a^	57.49^abc^	23.65^b^	50.64^abc^	47.09^ab^	56.17^ab^	70.49
10	90.21^a^	89.97^ab^	90.61^a^	87.85^a^	57.45^abc^	28.29^b^	48.72^abc^	53.79^ab^	56.83^ab^	68.01
11	90.05^ab^	89.95^ab^	90.57^ab^	90.44^a^	59.27^abc^	26.28^b^	48.93^abc^	50.07^ab^	56.13^ab^	69.52
12	89.76^ab^	89.43^ab^	90.03^ab^	89.95^a^	63.63^ab^	29.32^b^	48.62^abc^	44.31^ab^	52.72^ab^	68.27
13	90.77^a^	90.37^a^	91.06^a^	88.59^a^	61.35^ab^	31.83^b^	37.85^cd^	53.06^ab^	68.51^a^	76.4
14	88.38^ab^	88.22^ab^	88.90^ab^	89.06^a^	52.55^bc^	22.68^b^	48.61^abc^	44.86^ab^	45.71^b^	70.32
15	89.22^ab^	89.45^ab^	90.20^ab^	86.53^a^	62.09^ab^	29.30^b^	16.76^e^	57.13^a^	62.62^ab^	70.92
16	88.78^ab^	88.90^ab^	89.70^ab^	87.95^a^	60.11^ab^	30.90^b^	22.10^e^	50.91^ab^	54.00^ab^	68.18
17	89.46^ab^	89.62^ab^	90.44^ab^	87.71^a^	57.45^abc^	23.69^b^	10.88^e^	51.99^ab^	58.31^ab^	71.92
SEM	0.54	0.51	0.48	1.01	2.31	3.79	3.52	5.65	4.06	2.99
*p*-value	0.01	0.02	0.02	<0.01	<0.01	<0.01	<0.01	0.04	0.03	0.71

^a,b,c,d,e^Different letters in the same indicator represent significant differences, *n* = 6.

1 The information about sources of wheat is described in Table 1.

2 GE, gross energy; DM, dry matter; OM, organic matter; CP, crude protein; NDF, neutral detergent fiber.

### 3.3 Differences in the DE and ME contents of 17 samples of wheat

In the present study, the DE and ME contents ranged from 16.06 to 16.88 MJ/kg DM and 15.65 to 16.36 MJ/kg DM in 17 wheat cultivars, respectively (Table 7). In agreement with our findings, previous study demonstrated that the DE and ME contents were ranged from 16.40 to 17.02 MJ/kg DM and 15.73 to 16.49 MJ/kg DM in 12 wheat cultivars (12). Other research demonstrated that the DE of 5 wheat cultivars was from 16.19 to 16.99 MJ/kg DM (15). Similarly to the above researches, another study indicated that the DE content of 50 wheat cultivars (10 varieties each grown at five sites) were ranged from 15.49–16.95 MJ/kg (24). The DE content of No. 5 wheat sample is lowest, which may be due to its lower GE and CP content. On the other hand, high starch content of No. 5 wheat sample hindered the interaction between digestive enzymes and substrates (29, 30). The ATTD of CP, GE, and DM in No. 10 and 11 wheat samples were higher, and NDF content was lower, which may mainly contribute to greater DE and ME. However, in this study, we found that the DE content was higher than the values (13.44 to 13.71 MJ/kg) reported by Zhao et al. (14). This may due to higher ATTD of GE and EE content. Because fats facilitate digestion and reduce the flow rate of chyme (31), meanwhile, lower NDF content decreases endogenous losses of protein and fat, resulting in increasing of the excretion of organic matter (32, 33). In addition, carbohydrates are the largest source of energy for wheat, and carbohydrate digestibility largely determines the DE and ME contents of wheat (11). The discrepant results of the DE and ME contents may attribute to the differences in chemical composition and physical characteristics resulting from different cultivars and growing environment of wheat (34, 35) (see Table 8).

**Table 7:** The DE and ME of wheat samples.

Samples^1^	Items^2^				
	DE, MJ/kg As fed basis	ME, MJ/kg As fed basis	DE, MJ/kg DM basis	ME, MJ/kg DM basis	ME/DE
1	14.67^abc^	14.12	16.70^ab^	16.08	96.26
2	14.69^abc^	14.28	16.65^ab^	16.17	97.16
3	14.50^bc^	13.98	16.45^abc^	15.86	96.43
4	14.71^abc^	14.13	16.71^ab^	16.05	96.09
5	14.16^d^	13.80	16.06^c^	15.65	97.44
6	14.48^c^	14.13	16.39^abc^	15.98	97.51
7	14.51^bc^	14.07	16.47^abc^	15.97	96.98
8	14.64^abc^	14.08	16.53^ab^	15.90	96.22
9	14.81^abc^	14.16	16.62^ab^	15.90	95.67
10	15.03^a^	14.48	16.88^a^	16.26	96.35
11	14.98^a^	14.54	16.86^a^	16.36	97.01
12	14.68^abc^	14.04	16.61^ab^	15.88	95.57
13	14.94^ab^	14.50	16.83^ab^	16.34	97.09
14	14.54^bc^	14.02	16.44^abc^	15.86	96.43
15	14.63^abc^	14.17	16.52^ab^	16.00	96.83
16	14.58^abc^	14.28	16.41^abc^	16.07	97.96
17	14.74^abc^	14.28	16.35^bc^	15.84	96.89
SEM	0.09	0.15	0.10	0.16	0.95
*p*-value	<0.01	0.06	<0.01	0.21	0.96

^a,b,c^Different letters in the same indicator represent significant differences, *n* = 6.

1 The information about sources of wheat is described in Table 1.

2 DE, digestible energy; ME, metabolizable energy.

**Table 8:** Regression equations of DE and ME in wheat samples.

No.	Equations^1^	*R* ^2^	RMSE^2^	AIC^3^	BIC^4^	*p*-value
1	DE = 26.6394–0.6783 GE + 0.1618 CP	0.72	0.12	69.58	−63.48	<0.001
2	DE = 14.6609 + 0.1216 CP	0.65	0.13	−67.99	−64.22	<0.001
3	ME = −0.3869 + 0.7788 DE + 0.0336 starch + 0.0020 bulk density	0.81	0.09	−78.02	−73.52	<0.001
4	ME = 0.9999 + 0.8218 DE + 0.0229 starch	0.77	0.10	76.47	−73.96	<0.001
5	ME = 3.1923 + 0.7741 DE	0.73	0.10	−75.72	−74.03	<0.001

1 Regression equations were developed by stepwise regression analyses; DE, digestible energy; ME, metabolizable energy.

2 RMSE, root mean square error.

3 AIC, Akaike information criterion.

4 BIC, Bayesian information criterion.

### 3.4 The development of regression equations for DE and ME contents of wheat

**Figure 1:**
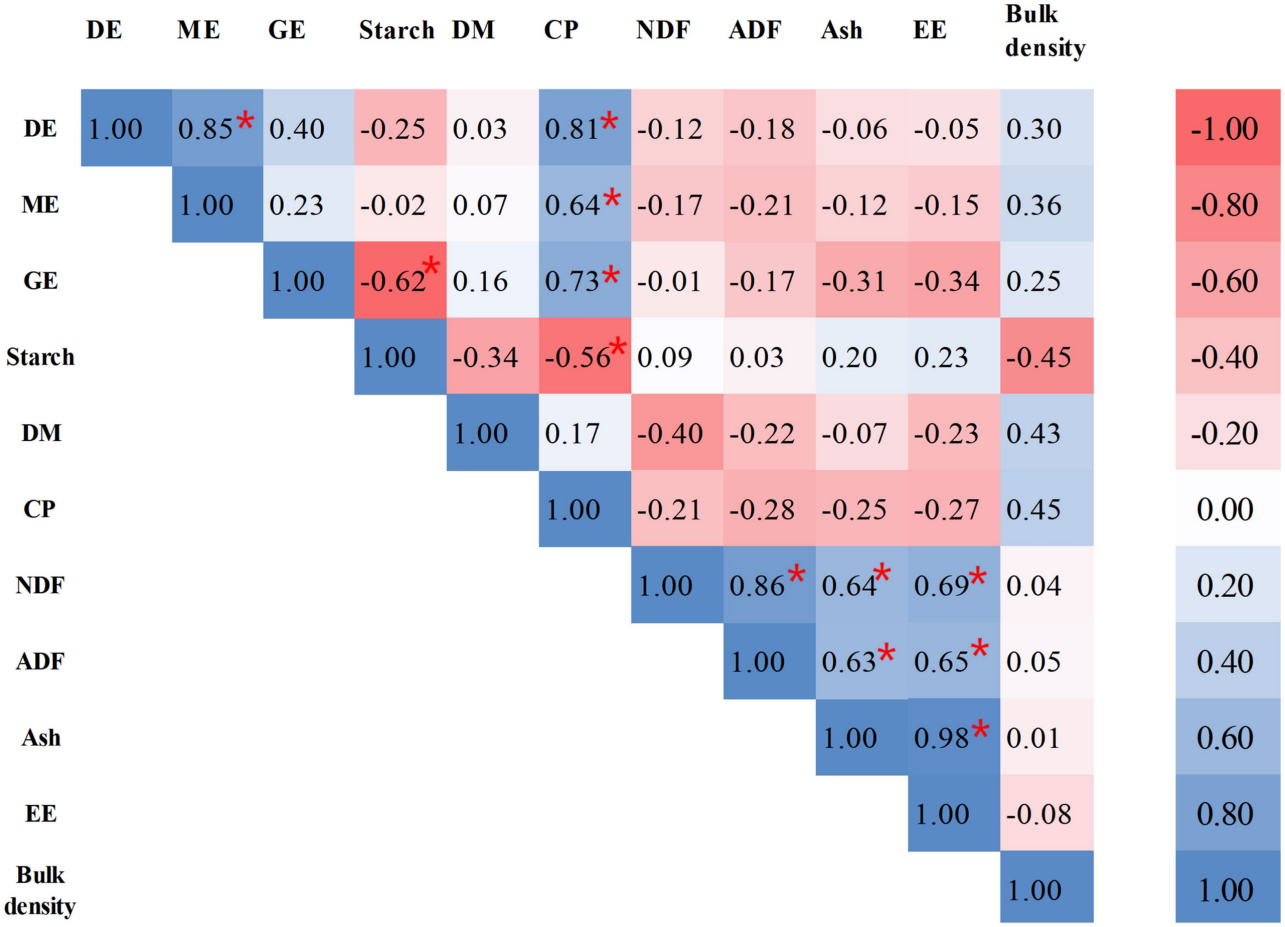
Correlation of DE, ME and chemical components of wheat samples. ^*^Represent *p* < 0.05. DE, digestible energy; ME, metabolizable energy; GE, gross energy; DM, dry matter; CP, crude protein; NDF, neutral detergent fiber; ADF, acid detergent fiber; EE, ether extract.

The metabolizable energy values derived from distinct wheat cultivars, heterogeneous experimental protocols, and divergent computational methodologies exhibit substantial variability. Regression models predicated on wheat chemical composition have been established as a recognized and standardized predictive paradigm. Previous studies have indicated that both ADF and NDF content are negatively correlated with DE of wheat, which is attributed to the reduced nutrient digestibility caused by the anti-nutritive properties of fibrous components (36, 37). Consequently, ADF and NDF are widely regarded as reliable predictors of available energy in wheat, due to their negative impact on the digestibility of nutrients (38, 39). Apart from macronutrients, wheat contains a diverse array of compounds such as phenolic acids, flavonoids, and carotenoids. Advanced analytical techniques can elucidate the variability in the content and composition of these compounds in different wheat varieties, growing environments, and processing methods (40). The variability of the compounds could be utilized to predict the nutritional quality and metabolic characteristics of wheat in animal diets. NDF and ADF contribute to a higher heat increment during fermentation or digestion processes and physically impede the digestion of other nutrients. Hence, they are routinely incorporated as negative predictors in net energy prediction equations (41). On the other hand, a prediction equation was developed by Son et al. (7) and Huang et al. (23) through nutritional composition analysis of the diet (including corn, soybean meal, and wheat), wherein NDF and ADF were identified as the principal predictive factors. Consequently, neutral detergent fiber (NDF) and acid detergent fiber (ADF) consistently serve as fundamental predictive variables in energy prediction models of diets with high fiber content. Previous research investigated the energy prediction model of high-fiber wheat bran and derived the prediction equation: NE = 11.16 − 0.09NDF (*R*^2^ = 0.71) and NE = 10.51 − 0.27ADF (*R*^2^ = 0.68), however, the regression equation is NE = −21.65 + 1.20 GE + 0.35 × CP (*R*^2^ = 0.94) and the *R*^2^ was elevated (0.65 vs. 0.94) when the GE and CP was regarded as predictors of regression equation (38). Therefore, CP and gross energy GE serve as the dominant predictors in energy prediction models. In this study, there were no significant correlations between ADF or NDF and DE content (Figure 1). This different results is likely because the samples were whole wheat grains in our study, which have a lower and less variable fiber content compared to bran. The limited range of fiber variation was insufficient to exert a dominant anti-nutritive effect on energy digestibility, allowing the positive effects of CP and GE to be the primary determinants. The result could be associated with a faint variation of NDF and ADF contents in wheat samples. In summary, ADF and NDF did not serve as predictive factors in our study.

GE was significantly negatively correlated with starch (*p* < 0.05). Compared to protein and fat, starch is the primary component in wheat with a relatively low energy density. When the starch content is high in wheat, the levels of other components are decline, particularly protein and fat, which has an extremely high GE. Meanwhile, the formation of starch-protein interactions contributes to reduction GE (42). The CP content was positively related (*p* < 0.05) to GE, DE, and ME concentrations in wheat. The most suitable predictor for DE was the combination of CP in the present study (Figure 1), which was consistent with a previous study that reported the variation of DE in different wheat samples was mainly caused by the content of CP (35). We analyzed data and gained a regression equation of DE = 14.6609 + 0.1216 CP. Previous research demonstrated that GE could precisely predict the DE content of wheat, and gained a regression equation of DE = −1,384 + 1.1 GE + 0.65 bulk weight (*R*^2^ = 0.83) (12). Thus, based on the equation, the GE was added as a predictor of regression equation on account of positive correlation between GE and CP content of wheat. We gained a regression equation of DE = 26.6394 − 0.6783 GE + 0.1618 CP and found that the *R*^2^ was elevated (0.65 vs. 0.72), and the analytical method referred to previous research (43). Differences in predictive factors and weighting coefficients could originate from seasonal variations and regional differences in wheat sample collection. The above findings validate the universality of crude protein, gross energy, and bulk density in predicting the energy value of wheat once again.

In this study, the most suitable predictor for ME was the DE content of wheat, which was consistent with some previous studies (44–46). In this study, regression equation is ME = 3.1923 + 0.7741 DE (*R*^2^ = 0.73). Huang et al. (23) demonstrated that regression equation is ME = 0.92 DE + 0.44 (*R*^2^ = 0.96) in mixed diet (include corn, soybean, and wheat shorts). In addition, Zhao et al. (14) found that regression equation is ME = 0.9716 × DE (MJ/kg) + 0.0636 (*R*^2^ = 0.99) in mixed diet (corn, wheat, and rice). The difference in *R*^2^ between the above results and our findings might be attributed to high corn content in diets. Corn, as a energy-dense ingredient with relatively uniform composition, minimizes variation in key predictors like starch content, delivering tighter statistical constraints and higher *R*^2^. Previous study reported starch was the main component for predicting energy metabolism (47). Thus, the starch and bulk density was added as predictors of regression equation in this study based on the above information. We gain regression equation of ME = −0.3869 + 0.7788 DE + 0.0336 starch + 0.0020 bulk density (*R*^2^ = 0.81). The prediction accuracy of ME was improved when starch and bulk density were incorporated into the prediction model as the second and third predictive factors.

## 4 Conclusion

There are apparent differences in the chemical constituents and energy contents among the 17 samples of wheat. The variability in available energy and nutrients digestibility of wheat samples was mainly associated with the differences in the chemical constituents. Chemical composition of wheat must be considered when wheat was used for formulating pig feeds. Predictive models of energy content were exploited. The optimal predictive model for the DE and ME were DE = 26.6394 − 0.6783 GE + 0.1618 CP and ME = −0.3869 + 0.7788 DE + 0.0336 starch + 0.0020 bulk density. Previous studies have demonstrated that the incorporation of wheat at a 60% inclusion rate in corn-soybean meal-based diets for pigs can reduce the usage of corn and soybean meal by approximately 55 and 7%, respectively, without compromising normal growth performance (2). The model have important practical significance in using wheat for precise diet configurations in pigs.

### 4.1 Potential future directions

Further research will focus on elucidating the interaction effect between fiber composition and other nutrients (e.g., arabinoxylans and β-glucans).

## Data Availability

The raw data supporting the conclusions of this article will be made available by the authors, without undue reservation.
